# Quetiapine *N*-oxide–fumaric acid (2/1)

**DOI:** 10.1107/S1600536812020818

**Published:** 2012-05-16

**Authors:** Jin Shen, Jing-Jing Qian, Su-Xiang Wu, Jian-Ming Gu, Xiu-Rong Hu

**Affiliations:** a College of Pharmaceutical Science, Zhejiang Chinese Medical University, Hangzhou, Zhejiang 310053, People’s Republic of China; b Chemistry Department, Zhejiang University, Hangzhou, Zhejiang 310028, People’s Republic of China

## Abstract

The title compound (systematic name: 2-{2-[4-(dibenzo[*b*,*f*][1,4]thia­zepin-11-yl)piperazin-1-yl 1-oxide]eth­oxy}ethanol–fumaric acid (2/1)), C_21_H_25_N_3_O_3_S·0.5C_4_H_4_O_4_, is one of the oxidation products of quetiapine hemifumaric acid. In the tricyclic fragment, the central thia­zepine ring displays a boat conformation and the benzene rings are inclined to each other at a dihedral angle of 72.0 (2)°. The piperazine ring adopts a chair conformation with its eth­oxy­ethanol side chain oriented equatorially. In addition to the main mol­ecule, the asymmetric unit contains one-half mol­ecule of fumaric acid, the complete mol­ecule being generated by inversion symmetry. In the crystal, O—H⋯O hydrogen bonds link the components into corrugated layers parallel to *bc* plane.

## Related literature

For the identification, isolation, synthesis and characterization of quetiapine *N*-oxide, see: Mittapelli *et al.* (2010 ▶). For quanti­tative determination of quetiapine impurities, degradation products in pharmaceutical dosage form or in bulk, tablets, and in human plasma, see: Trivedi & Patel (2011 ▶); Belal *et al.* (2008 ▶). For the use of quetiapine as an anti­psychotic drug, see: Lieberman (1996 ▶). For the crystal structure of quetiapine hemifumarate, see: Ravikumar & Sridhar (2005 ▶).
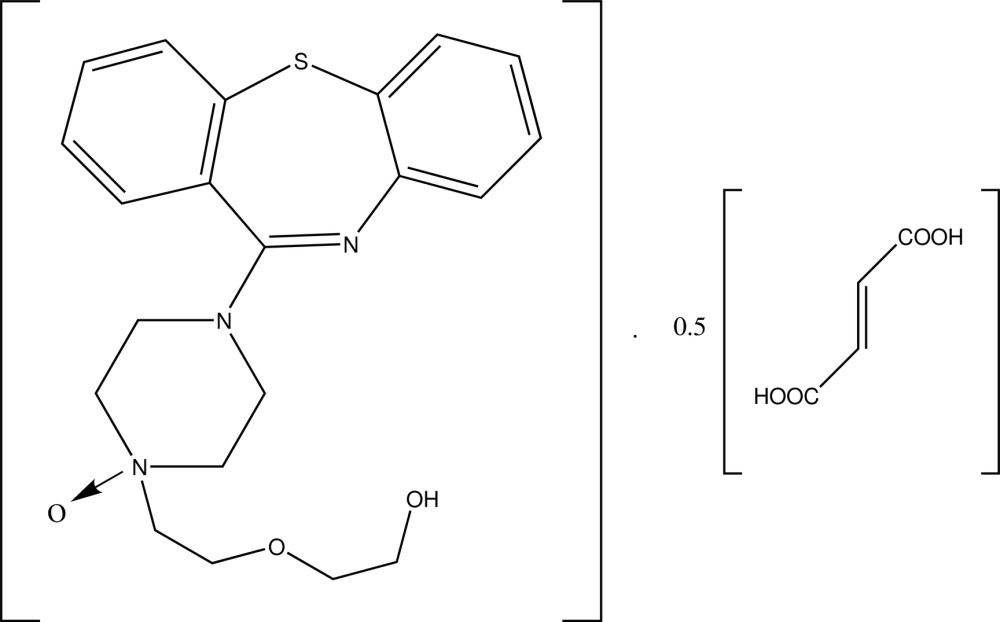


## Experimental

### 

#### Crystal data

C_21_H_25_N_3_O_3_S·0.5C_4_H_4_O_4_
*M* *_r_* = 457.54
Monoclinic, 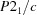

*a* = 13.1299 (9) Å
*b* = 12.5047 (8) Å
*c* = 13.9950 (9) Å
β = 101.59 (2)°
*V* = 2250.9 (3) Å^3^
*Z* = 4
Mo *K*α radiation
μ = 0.18 mm^−1^
*T* = 296 K
0.27 × 0.25 × 0.10 mm

#### Data collection

Rigaku R-AXIS RAPID/ZJUG diffractometer
Absorption correction: multi-scan (*ABSCOR*; Higashi, 1995 ▶) *T*_min_ = 0.947, *T*_max_ = 0.982
17003 measured reflections
3970 independent reflections
2453 reflections with *I* > 2σ(*I*)
*R*_int_ = 0.069

#### Refinement

*R*[*F* ^2^ > 2σ(*F* ^2^)] = 0.070
*wR*(*F* ^2^) = 0.193
*S* = 1.00
3970 reflections
291 parameters
H-atom parameters constrained
Δρ_max_ = 0.37 e Å^−3^
Δρ_min_ = −0.39 e Å^−3^

### 

Data collection: *PROCESS-AUTO* (Rigaku, 2006 ▶); cell refinement: *PROCESS-AUTO*; data reduction: *CrystalStructure* (Rigaku, 2007 ▶); program(s) used to solve structure: *SHELXS97* (Sheldrick, 2008 ▶); program(s) used to refine structure: *SHELXL97* (Sheldrick, 2008 ▶); molecular graphics: *ORTEP-3 for Windows* (Farrugia, 1997 ▶); software used to prepare material for publication: *WinGX* (Farrugia, 1999 ▶).

## Supplementary Material

Crystal structure: contains datablock(s) global, I. DOI: 10.1107/S1600536812020818/cv5294sup1.cif

Structure factors: contains datablock(s) I. DOI: 10.1107/S1600536812020818/cv5294Isup2.hkl

Additional supplementary materials:  crystallographic information; 3D view; checkCIF report

## Figures and Tables

**Table 1:** Hydrogen-bond geometry (Å, °)

*D*—H⋯*A*	*D*—H	H⋯*A*	*D*⋯*A*	*D*—H⋯*A*
O4—H401⋯O1	0.81	1.62	2.394 (6)	157
O3—H301⋯O1^i^	0.82	1.90	2.691 (4)	161

Symmetry code: (i) 
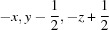
.

**Fig. 1.**
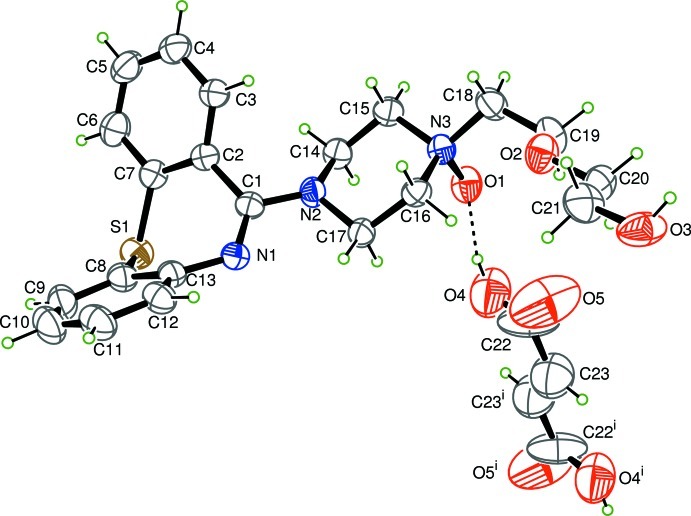
View of (I) showing atom-labelling scheme and 40% probability displacement ellipsoids. H atoms are shown as small circles of arbitary radii. Dashed line denotes hydrogen bond.

## supplementary crystallographic information

### Comment

Quetiapine N-oxide hemifumarate is one of the oxidation or degradation products of quetiapine hemifumarate (Mittapelli *et al.*, 2010; Trivedi *et al.*, 2011 & Belal *et al.*, 2008). Quetiapine is one of the atypical antipsychotic licensed for the treatment of schizophrenia (Lieberman, 1996) or manic episodes associated with bipolar disorder. In the present study, we report the crystal structure of quetiapine N-oxide hemifumarate, (I), recrystallized from ethanol.

In the crystal structure of (I) (Fig.1), the asymmetric unit consists of one quetiapine N-oxide molecule and one-half of fumarate molecule; the latter one is situated on inversion center. The oxidized N atom is established as N3. The N—C bonds at N3 are lengthened [mean value 1.504 (5) Å compared to 1.427 (5) Å for N2], as would be expected for an oxidized system. The values of bond length for N3—O1 is 1.388 (4) Å. Consequently, N3 shows quaternary character in a tetrahedral configuration, with bond angles ranging from 108.5 (3)° to 110.3 (3)°.

The conformation of the title compound is similar to that of quetiapine hemifumarate (Ravikumar *et al.*, 2005). The conformation of the central thiazepine ring in the (6,7,6)-tricyclic ring system can be described as a boat, with the atoms common to the benzene rings (C2, C7, C8 and C13) as the basal plane, the S atom as the bow and the N1=C1 bridge as the stern. The bow angle is 50.0 (2)° and the stern angle is 41.7 (2)°. This enables the dibenzothiazepine ring skeleton to form a flattened V-shaped conformation. The dihedral angle between the two benzene rings is 72.0 (2)°. The piperazine ring adopts a chair conformation. The thiazepine nucleus can be viewed as being in an equatorial orientation to the piperazine ring. The ethoxyethanol side chain at the oxidized N-atom site of the piperazine ring occupies an equatorial orientation and is in a folded conformation.

In the crystal structure, intermolecular hydrogen bonds O—H···O (Table 1) link all moieties into corrugated layers parallel to *bc* plane.

### Experimental

The crude product synthesized by reacting quetiapine hemifumarate with hydrogen peroxideis was supplied by Zhejiang Supor Pharmaceuticals Co., Ltd. It was recrystallized from ethanol solution, giving colourless crystals of (I) suitable for X-ray diffraction.

### Refinement

The H atoms were placed in calculated positions [O—H 0.82 Å; C—H 0.93–0.97 Å] and refinded as riding, with *U*_iso_(H) = 1.2–1.5*U*_eq_ (carrier atom).

### Crystal data

**Table:**

C_21_H_25_N_3_O_3_S·0.5C_4_H_4_O_4_	*F*(000) = 968
*M**_r_* = 457.54	*D*_x_ = 1.350 Mg m^−^^3^
Monoclinic, *P*2_1_/*c*	Mo *K*α radiation, λ = 0.71073 Å
Hall symbol: -P 2ybc	Cell parameters from 10875 reflections
*a* = 13.1299 (9) Å	θ = 3.1–27.5°
*b* = 12.5047 (8) Å	µ = 0.18 mm^−^^1^
*c* = 13.9950 (9) Å	*T* = 296 K
β = 101.59 (2)°	Platelet, colourless
*V* = 2250.9 (3) Å^3^	0.27 × 0.25 × 0.10 mm
*Z* = 4	

### Data collection

**Table:**

Rigaku R-AXIS RAPID/ZJUG diffractometer	3970 independent reflections
Radiation source: rolling anode	2453 reflections with *I* > 2σ(*I*)
Graphite monochromator	*R*_int_ = 0.069
Detector resolution: 10.00 pixels mm^-1^	θ_max_ = 25.0°, θ_min_ = 3.1°
ω scans	*h* = −15→15
Absorption correction: multi-scan (*ABSCOR*; Higashi, 1995)	*k* = −14→14
*T*_min_ = 0.947, *T*_max_ = 0.982	*l* = −16→16
17003 measured reflections	

### Refinement

**Table:**

Refinement on *F*^2^	Secondary atom site location: difference Fourier map
Least-squares matrix: full	Hydrogen site location: inferred from neighbouring sites
*R*[*F*^2^ > 2σ(*F*^2^)] = 0.070	H-atom parameters constrained
*wR*(*F*^2^) = 0.193	*w* = 1/[σ^2^(*F*_o_^2^) + (0.0701*P*)^2^ + 3.2854*P*] where *P* = (*F*_o_^2^ + 2*F*_c_^2^)/3
*S* = 1.00	(Δ/σ)_max_ < 0.001
3970 reflections	Δρ_max_ = 0.37 e Å^−^^3^
291 parameters	Δρ_min_ = −0.39 e Å^−^^3^
0 restraints	Extinction correction: *SHELXL97* (Sheldrick, 2008), Fc^*^=kFc[1+0.001xFc^2^λ^3^/sin(2θ)]^-1/4^
Primary atom site location: structure-invariant direct methods	Extinction coefficient: 0.0142 (18)

### Special details

**Table:**

Geometry. All e.s.d.'s (except the e.s.d. in the dihedral angle between two l.s. planes) are estimated using the full covariance matrix. The cell e.s.d.'s are taken into account individually in the estimation of e.s.d.'s in distances, angles and torsion angles; correlations between e.s.d.'s in cell parameters are only used when they are defined by crystal symmetry. An approximate (isotropic) treatment of cell e.s.d.'s is used for estimating e.s.d.'s involving l.s. planes.
Refinement. Refinement of *F*^2^ against ALL reflections. The weighted *R*-factor *wR* and goodness of fit *S* are based on *F*^2^, conventional *R*-factors *R* are based on *F*, with *F* set to zero for negative *F*^2^. The threshold expression of *F*^2^ > σ(*F*^2^) is used only for calculating *R*-factors(gt) *etc*. and is not relevant to the choice of reflections for refinement. *R*-factors based on *F*^2^ are statistically about twice as large as those based on *F*, and *R*- factors based on ALL data will be even larger.

### Fractional atomic coordinates and isotropic or equivalent isotropic displacement parameters (Å^2^)

**Table:**

	*x*	*y*	*z*	*U*_iso_*/*U*_eq_	
C1	0.4958 (3)	0.6665 (3)	0.5761 (3)	0.0441 (9)	
C2	0.6017 (3)	0.6567 (3)	0.5537 (3)	0.0433 (9)	
C3	0.6156 (3)	0.6002 (3)	0.4723 (3)	0.0490 (10)	
H3	0.5591	0.5665	0.4332	0.059*	
C4	0.7121 (4)	0.5933 (3)	0.4486 (3)	0.0586 (11)	
H4	0.7205	0.5554	0.3936	0.070*	
C5	0.7957 (4)	0.6424 (4)	0.5060 (4)	0.0688 (13)	
H5	0.8601	0.6407	0.4880	0.083*	
C6	0.7850 (4)	0.6941 (4)	0.5900 (4)	0.0648 (12)	
H6	0.8430	0.7235	0.6306	0.078*	
C7	0.6881 (3)	0.7027 (3)	0.6146 (3)	0.0486 (10)	
C8	0.6330 (3)	0.6545 (3)	0.7814 (3)	0.0522 (10)	
C9	0.6911 (4)	0.6175 (4)	0.8694 (3)	0.0683 (13)	
H9	0.7536	0.6507	0.8964	0.082*	
C10	0.6572 (4)	0.5327 (4)	0.9169 (3)	0.0775 (15)	
H10	0.6963	0.5090	0.9760	0.093*	
C11	0.5653 (4)	0.4833 (4)	0.8767 (3)	0.0718 (14)	
H11	0.5429	0.4249	0.9082	0.086*	
C12	0.5060 (4)	0.5190 (3)	0.7908 (3)	0.0564 (11)	
H12	0.4430	0.4858	0.7655	0.068*	
C13	0.5391 (3)	0.6045 (3)	0.7405 (3)	0.0477 (10)	
C14	0.4310 (3)	0.7560 (3)	0.4149 (3)	0.0501 (10)	
H14A	0.5038	0.7567	0.4103	0.060*	
H14B	0.4092	0.8292	0.4217	0.060*	
C15	0.3672 (3)	0.7076 (3)	0.3242 (3)	0.0519 (10)	
H15A	0.3912	0.6354	0.3162	0.062*	
H15B	0.3760	0.7491	0.2679	0.062*	
C16	0.2418 (3)	0.6465 (3)	0.4204 (3)	0.0472 (10)	
H16A	0.2592	0.5718	0.4142	0.057*	
H16B	0.1697	0.6503	0.4269	0.057*	
C17	0.3100 (3)	0.6920 (3)	0.5109 (3)	0.0491 (10)	
H17A	0.2876	0.7641	0.5220	0.059*	
H17B	0.3037	0.6487	0.5670	0.059*	
C18	0.1943 (3)	0.6514 (4)	0.2394 (3)	0.0634 (12)	
H18A	0.2070	0.6898	0.1827	0.076*	
H18B	0.2207	0.5793	0.2364	0.076*	
C19	0.0792 (4)	0.6461 (4)	0.2346 (3)	0.0675 (13)	
H19A	0.0563	0.7106	0.2625	0.081*	
H19B	0.0431	0.6413	0.1671	0.081*	
C21	−0.0675 (4)	0.4476 (4)	0.3412 (4)	0.0770 (15)	
H21A	−0.0322	0.3870	0.3192	0.092*	
H21B	−0.0360	0.4607	0.4090	0.092*	
C22	0.0380 (8)	0.8697 (5)	0.4497 (6)	0.119 (3)	
C23	−0.0191 (6)	0.9488 (5)	0.4871 (6)	0.119 (2)	
H23	−0.0859	0.9320	0.4952	0.142*	
C20	−0.0523 (3)	0.5435 (4)	0.2823 (4)	0.0687 (13)	
H20A	−0.0884	0.5340	0.2152	0.082*	
H20B	−0.0802	0.6065	0.3084	0.082*	
N1	0.4694 (2)	0.6414 (3)	0.6578 (2)	0.0464 (8)	
N2	0.4178 (2)	0.6936 (3)	0.4998 (2)	0.0474 (8)	
N3	0.2541 (2)	0.7051 (2)	0.3299 (2)	0.0464 (8)	
O1	0.2203 (2)	0.8100 (2)	0.3328 (2)	0.0617 (8)	
O2	0.0553 (2)	0.5560 (2)	0.2866 (2)	0.0664 (9)	
O3	−0.1721 (3)	0.4219 (3)	0.3344 (3)	0.0910 (12)	
H301	−0.1962	0.3992	0.2796	0.136*	
O4	0.1258 (4)	0.8974 (4)	0.4401 (4)	0.1291 (18)	
H401	0.1599	0.8556	0.4147	0.194*	
O5	0.0062 (6)	0.7872 (6)	0.4257 (6)	0.195 (3)	
S1	0.67581 (9)	0.76727 (9)	0.72389 (9)	0.0615 (4)	

### Atomic displacement parameters (Å^2^)

**Table:**

	*U*^11^	*U*^22^	*U*^33^	*U*^12^	*U*^13^	*U*^23^
C1	0.049 (2)	0.039 (2)	0.044 (2)	−0.0020 (17)	0.0103 (18)	−0.0027 (17)
C2	0.048 (2)	0.038 (2)	0.045 (2)	0.0006 (17)	0.0131 (18)	0.0029 (17)
C3	0.055 (3)	0.045 (2)	0.048 (2)	0.0017 (19)	0.0126 (19)	0.0013 (19)
C4	0.068 (3)	0.053 (3)	0.059 (3)	0.015 (2)	0.022 (2)	0.011 (2)
C5	0.055 (3)	0.081 (3)	0.075 (3)	0.015 (3)	0.025 (2)	0.012 (3)
C6	0.049 (3)	0.068 (3)	0.076 (3)	−0.003 (2)	0.011 (2)	0.004 (3)
C7	0.047 (2)	0.046 (2)	0.052 (2)	−0.0020 (18)	0.0093 (18)	−0.0001 (18)
C8	0.058 (3)	0.048 (2)	0.050 (2)	0.004 (2)	0.008 (2)	−0.0079 (19)
C9	0.069 (3)	0.069 (3)	0.058 (3)	0.014 (2)	−0.008 (2)	−0.010 (2)
C10	0.100 (4)	0.073 (3)	0.052 (3)	0.024 (3)	−0.003 (3)	0.008 (3)
C11	0.102 (4)	0.059 (3)	0.053 (3)	0.013 (3)	0.012 (3)	0.009 (2)
C12	0.070 (3)	0.050 (2)	0.049 (2)	0.000 (2)	0.011 (2)	0.002 (2)
C13	0.054 (3)	0.047 (2)	0.042 (2)	0.0098 (19)	0.0086 (18)	−0.0052 (18)
C14	0.051 (2)	0.050 (2)	0.050 (2)	−0.0017 (19)	0.0119 (18)	0.0117 (19)
C15	0.052 (3)	0.057 (3)	0.049 (2)	−0.002 (2)	0.0166 (19)	0.0071 (19)
C16	0.047 (2)	0.048 (2)	0.049 (2)	0.0000 (18)	0.0159 (18)	0.0054 (18)
C17	0.041 (2)	0.062 (2)	0.045 (2)	0.0010 (19)	0.0085 (17)	0.0048 (19)
C18	0.060 (3)	0.079 (3)	0.049 (2)	−0.014 (2)	0.006 (2)	−0.007 (2)
C19	0.065 (3)	0.070 (3)	0.060 (3)	−0.012 (2)	−0.003 (2)	0.004 (2)
C21	0.078 (4)	0.069 (3)	0.083 (3)	−0.025 (3)	0.014 (3)	−0.020 (3)
C22	0.200 (9)	0.055 (4)	0.130 (6)	−0.020 (5)	0.103 (6)	−0.027 (4)
C23	0.113 (6)	0.108 (5)	0.133 (6)	0.004 (5)	0.018 (4)	−0.013 (5)
C20	0.050 (3)	0.065 (3)	0.090 (4)	−0.011 (2)	0.012 (2)	−0.018 (3)
N1	0.048 (2)	0.0485 (19)	0.0425 (18)	0.0038 (15)	0.0094 (15)	−0.0007 (15)
N2	0.0392 (18)	0.059 (2)	0.0435 (18)	0.0003 (15)	0.0064 (14)	0.0105 (15)
N3	0.049 (2)	0.0425 (18)	0.0467 (18)	0.0004 (15)	0.0072 (15)	0.0051 (14)
O1	0.065 (2)	0.0447 (16)	0.0709 (19)	0.0053 (14)	0.0028 (15)	0.0099 (14)
O2	0.0531 (19)	0.0605 (19)	0.083 (2)	−0.0114 (15)	0.0065 (15)	−0.0023 (17)
O3	0.087 (3)	0.091 (3)	0.108 (3)	−0.033 (2)	0.051 (2)	−0.045 (2)
O4	0.103 (4)	0.108 (4)	0.163 (5)	0.011 (3)	−0.003 (3)	−0.033 (3)
O5	0.197 (7)	0.132 (5)	0.292 (9)	−0.037 (5)	0.133 (6)	−0.052 (6)
S1	0.0640 (8)	0.0544 (7)	0.0645 (7)	−0.0083 (5)	0.0087 (5)	−0.0151 (6)

### Geometric parameters (Å, º)

**Table:**

C1—N1	1.299 (5)	C15—H15A	0.9700
C1—N2	1.366 (5)	C15—H15B	0.9700
C1—C2	1.491 (5)	C16—N3	1.500 (5)
C2—C3	1.384 (5)	C16—C17	1.508 (5)
C2—C7	1.398 (5)	C16—H16A	0.9700
C3—C4	1.375 (6)	C16—H16B	0.9700
C3—H3	0.9300	C17—N2	1.455 (5)
C4—C5	1.368 (6)	C17—H17A	0.9700
C4—H4	0.9300	C17—H17B	0.9700
C5—C6	1.374 (6)	C18—C19	1.501 (6)
C5—H5	0.9300	C18—N3	1.507 (5)
C6—C7	1.387 (6)	C18—H18A	0.9700
C6—H6	0.9300	C18—H18B	0.9700
C7—S1	1.765 (4)	C19—O2	1.410 (5)
C8—C9	1.390 (6)	C19—H19A	0.9700
C8—C13	1.398 (6)	C19—H19B	0.9700
C8—S1	1.771 (4)	C21—O3	1.394 (5)
C9—C10	1.372 (7)	C21—C20	1.491 (7)
C9—H9	0.9300	C21—H21A	0.9700
C10—C11	1.372 (7)	C21—H21B	0.9700
C10—H10	0.9300	C22—O5	1.138 (8)
C11—C12	1.370 (6)	C22—O4	1.237 (8)
C11—H11	0.9300	C22—C23	1.404 (9)
C12—C13	1.397 (6)	C23—C23^i^	1.396 (13)
C12—H12	0.9300	C23—H23	0.9300
C13—N1	1.401 (5)	C20—O2	1.411 (5)
C14—N2	1.461 (5)	C20—H20A	0.9700
C14—C15	1.501 (5)	C20—H20B	0.9700
C14—H14A	0.9700	N3—O1	1.388 (4)
C14—H14B	0.9700	O3—H301	0.8200
C15—N3	1.504 (5)	O4—H401	0.8139
			
N1—C1—N2	117.2 (4)	N3—C16—H16B	109.1
N1—C1—C2	126.2 (3)	C17—C16—H16B	109.1
N2—C1—C2	116.2 (3)	H16A—C16—H16B	107.9
C3—C2—C7	119.2 (4)	N2—C17—C16	109.9 (3)
C3—C2—C1	119.8 (3)	N2—C17—H17A	109.7
C7—C2—C1	121.0 (3)	C16—C17—H17A	109.7
C4—C3—C2	120.7 (4)	N2—C17—H17B	109.7
C4—C3—H3	119.6	C16—C17—H17B	109.7
C2—C3—H3	119.6	H17A—C17—H17B	108.2
C5—C4—C3	120.0 (4)	C19—C18—N3	114.0 (4)
C5—C4—H4	120.0	C19—C18—H18A	108.8
C3—C4—H4	120.0	N3—C18—H18A	108.8
C4—C5—C6	120.4 (4)	C19—C18—H18B	108.8
C4—C5—H5	119.8	N3—C18—H18B	108.8
C6—C5—H5	119.8	H18A—C18—H18B	107.7
C5—C6—C7	120.4 (4)	O2—C19—C18	109.8 (4)
C5—C6—H6	119.8	O2—C19—H19A	109.7
C7—C6—H6	119.8	C18—C19—H19A	109.7
C6—C7—C2	119.2 (4)	O2—C19—H19B	109.7
C6—C7—S1	119.9 (3)	C18—C19—H19B	109.7
C2—C7—S1	120.8 (3)	H19A—C19—H19B	108.2
C9—C8—C13	119.7 (4)	O3—C21—C20	112.8 (5)
C9—C8—S1	120.0 (4)	O3—C21—H21A	109.0
C13—C8—S1	120.2 (3)	C20—C21—H21A	109.0
C10—C9—C8	120.8 (5)	O3—C21—H21B	109.0
C10—C9—H9	119.6	C20—C21—H21B	109.0
C8—C9—H9	119.6	H21A—C21—H21B	107.8
C11—C10—C9	119.6 (4)	O5—C22—O4	121.1 (8)
C11—C10—H10	120.2	O5—C22—C23	123.8 (9)
C9—C10—H10	120.2	O4—C22—C23	115.0 (7)
C12—C11—C10	120.7 (5)	C23^i^—C23—C22	123.5 (10)
C12—C11—H11	119.7	C23^i^—C23—H23	118.3
C10—C11—H11	119.7	C22—C23—H23	118.3
C11—C12—C13	120.9 (4)	O2—C20—C21	108.1 (4)
C11—C12—H12	119.6	O2—C20—H20A	110.1
C13—C12—H12	119.6	C21—C20—H20A	110.1
C12—C13—C8	118.2 (4)	O2—C20—H20B	110.1
C12—C13—N1	116.8 (4)	C21—C20—H20B	110.1
C8—C13—N1	124.6 (4)	H20A—C20—H20B	108.4
N2—C14—C15	109.5 (3)	C1—N1—C13	124.2 (3)
N2—C14—H14A	109.8	C1—N2—C17	120.4 (3)
C15—C14—H14A	109.8	C1—N2—C14	125.1 (3)
N2—C14—H14B	109.8	C17—N2—C14	111.9 (3)
C15—C14—H14B	109.8	O1—N3—C16	110.3 (3)
H14A—C14—H14B	108.2	O1—N3—C15	107.9 (3)
C14—C15—N3	110.7 (3)	C16—N3—C15	109.2 (3)
C14—C15—H15A	109.5	O1—N3—C18	109.3 (3)
N3—C15—H15A	109.5	C16—N3—C18	111.4 (3)
C14—C15—H15B	109.5	C15—N3—C18	108.5 (3)
N3—C15—H15B	109.5	C19—O2—C20	113.1 (4)
H15A—C15—H15B	108.1	C21—O3—H301	109.5
N3—C16—C17	112.3 (3)	C22—O4—H401	118.3
N3—C16—H16A	109.1	C7—S1—C8	97.02 (19)
C17—C16—H16A	109.1		
			
N1—C1—C2—C3	125.9 (4)	O4—C22—C23—C23^i^	−0.5 (15)
N2—C1—C2—C3	−46.7 (5)	O3—C21—C20—O2	−174.3 (4)
N1—C1—C2—C7	−53.2 (6)	N2—C1—N1—C13	175.3 (3)
N2—C1—C2—C7	134.2 (4)	C2—C1—N1—C13	2.7 (6)
C7—C2—C3—C4	−3.0 (6)	C12—C13—N1—C1	−137.1 (4)
C1—C2—C3—C4	177.8 (4)	C8—C13—N1—C1	50.3 (6)
C2—C3—C4—C5	0.2 (6)	N1—C1—N2—C17	−1.1 (5)
C3—C4—C5—C6	3.3 (7)	C2—C1—N2—C17	172.2 (3)
C4—C5—C6—C7	−3.9 (7)	N1—C1—N2—C14	158.9 (4)
C5—C6—C7—C2	1.0 (7)	C2—C1—N2—C14	−27.8 (5)
C5—C6—C7—S1	178.8 (4)	C16—C17—N2—C1	−139.4 (4)
C3—C2—C7—C6	2.4 (6)	C16—C17—N2—C14	58.1 (4)
C1—C2—C7—C6	−178.4 (4)	C15—C14—N2—C1	137.9 (4)
C3—C2—C7—S1	−175.4 (3)	C15—C14—N2—C17	−60.6 (4)
C1—C2—C7—S1	3.7 (5)	C17—C16—N3—O1	−64.5 (4)
C13—C8—C9—C10	−0.2 (7)	C17—C16—N3—C15	54.0 (4)
S1—C8—C9—C10	177.5 (4)	C17—C16—N3—C18	173.9 (3)
C8—C9—C10—C11	0.5 (7)	C14—C15—N3—O1	64.2 (4)
C9—C10—C11—C12	−1.3 (8)	C14—C15—N3—C16	−55.8 (4)
C10—C11—C12—C13	1.8 (7)	C14—C15—N3—C18	−177.5 (3)
C11—C12—C13—C8	−1.5 (6)	C19—C18—N3—O1	−61.1 (5)
C11—C12—C13—N1	−174.6 (4)	C19—C18—N3—C16	61.1 (5)
C9—C8—C13—C12	0.7 (6)	C19—C18—N3—C15	−178.6 (4)
S1—C8—C13—C12	−177.0 (3)	C18—C19—O2—C20	−177.8 (4)
C9—C8—C13—N1	173.3 (4)	C21—C20—O2—C19	−178.9 (4)
S1—C8—C13—N1	−4.5 (5)	C6—C7—S1—C8	−116.3 (4)
N2—C14—C15—N3	59.1 (4)	C2—C7—S1—C8	61.6 (4)
N3—C16—C17—N2	−55.0 (4)	C9—C8—S1—C7	119.6 (4)
N3—C18—C19—O2	−84.5 (5)	C13—C8—S1—C7	−62.6 (4)
O5—C22—C23—C23^i^	−177.4 (11)		

Symmetry code: (i) −*x*, −*y*+2, −*z*+1.

### Hydrogen-bond geometry (Å, º)

**Table:**

*D*—H···*A*	*D*—H	H···*A*	*D*···*A*	*D*—H···*A*
O4—H401···O1	0.81	1.62	2.394 (6)	157
O3—H301···O1^ii^	0.82	1.90	2.691 (4)	161

Symmetry code: (ii) −*x*, *y*−1/2, −*z*+1/2.
